# Lysergic acid diethylamide-derived excitatory/inhibitory ratio change enhances global synchrony in functional brain dynamics

**DOI:** 10.1371/journal.pcbi.1013822

**Published:** 2025-12-15

**Authors:** Lingyu Zhang, Weiyang Shi, Ziyang Zhao, Zhichao Wang, Congying Chu, Bokai Zhao, Jiaqi Zhang, Qianhui Liu, Yueheng Lan, Tianzi Jiang

**Affiliations:** 1 School of Science, Beijing University of Posts and Telecommunications, Beijing, China; 2 Beijing Key Laboratory of Brainnetome and Brain-Computer Interface, Institute of Automation, Chinese Academy of Sciences, Beijing, China; 3 Brainnetome Center, Institute of Automation, Chinese Academy of Sciences, Beijing, China; 4 Gansu Provincial Key Laboratory of Wearable Computing, School of Information Science and Engineering, Lanzhou University, Gansu, China; 5 Nanhu Brain-computer Interface Institute, Hangzhou, China; 6 Institute of Neuroscience and Medicine (INM-2), Forschungszentrum Jülich, Jülich, Germany; 7 University of Chinese Academy of Sciences, Beijing, China; 8 Key Laboratory of Mathematics and Information Networks (Beijing University of Posts and Telecommunications), Ministry of Education, Beijing, China; 9 Xiaoxiang Institute for Brain Health and Yongzhou Central Hospital, Yongzhou, Hunan Province, China; ShanghaiTech University, CHINA

## Abstract

Lysergic acid diethylamide (LSD) has shown remarkable potential in modulating brain functional organization and dynamics. However, the exact mechanisms underlying its effects remain unclear. In this study, we employed a data-driven approach to analyze recurrent functional connectivity patterns in resting-state fMRI data and developed a parameterized feedback inhibition model to characterize excitatory/inhibitory (E/I) balance. The findings demonstrate that LSD enhances global brain synchrony and dynamic complexity. This enhanced synchrony likely stems from LSD’s preferential stabilization of a globally synchronized yet functionally non-modular brain state - a pattern showing higher occurrence probability and acts as an “attractor” that recruits transitions from cognitive control networks. Crucially, these phenomena appear underpinned by LSD-induced convergence of excitatory/inhibitory balance across cortical hierarchies, particularly through Sensorimotor (SOM) suppression coupled with transmodal potentiation, where the Sensorimotor cortices emerge as potential regulatory hubs driving this neurochemical rebalancing. These convergent effects are consistent with the emergence of a brain state characterized by weakened sensory anchoring and enhanced cognitive flexibility, where the typical separation between concrete perception and abstract cognition becomes blurred. This neurophysiological remodeling therefore suggests a potential mechanism that could contribute to LSD’s hallucinatory effects and its therapeutic potential in mental disorders characterized by rigid thought patterns.

## Author summary

This study provides new insights into the neurodynamic effects of LSD, highlighting its capacity to induce complex reorganization within brain networks by modulating the excitatory/inhibitory (E/I) balance. Our findings identify a mechanism—the leveling of the E/I balance gradient between sensorimotor and association cortices by LSD—that is associated with neurodynamic patterns interpreted as enhanced cognitive flexibility and suppressed sensory responses. This neurochemical reconfiguration predisposes the brain to enter a globally synchronized yet functionally non-modular metastable state. These effects may underlie LSD’s therapeutic potential for disrupting maladaptive patterns entrenched in mental disorders. Furthermore, we identify the SOM cortices as a driving area in mediating these processes, offering a promising target for future research into precision interventions.

## Introduction

Recently, interest in Lysergic acid diethylamide (LSD) and other psychedelics has resurged, spurred by clinical trials showing their potential for treating depression, anxiety, and addiction [1–3]. Research has shown that LSD significantly alters the brain functional organization and dynamics, including increased global brain network integration and enhanced connectivity between different networks [4]. Concurrently, functional brain network integrity decreases [5]. Furthermore, the repertoire of dynamical brain states expanded, as evidenced by increased variance in blood-oxygen-level-dependent (BOLD) signals [6], elevated entropy at both regional and global levels [7,8], and increased diversity in dynamic functional connectivity states [9]. These neurodynamic alterations reveal a characteristic LSD-induced reorganization of brain network architecture. This pharmacological remodeling presents a neurodynamic counterpoint to the rigid, pathologically entrenched states observed across various mental disorders. While existing findings elucidate what LSD does to brain networks, effective clinical translation requires deeper understanding of how it achieves these effects, particularly identifying the key driving regions. Such insights could lead to targeted strategies to disrupt maladaptive patterns entrenched in mental disorders, akin to using pharmacological “keys” to unlock pathological brain states.

Conventional methods of brain function analysis often focus on assessing the temporal correlation of activity across spatially separated brain regions, commonly referred to as functional connectivity [10,11]. However, recent research has highlighted that static analysis methods may not fully capture the dynamic changes in brain states [12–14]. This is because brain activity patterns in states like the psychedelic state are not static but involve dynamic interactions between different brain regions, exhibiting characteristic spatio-temporal dynamics [15,16]. Increasing evidence suggests that finer-grained, dynamic analytical approaches can provide deeper insights into the relationship between brain activity and behavioral states [17–19]. The Leading Eigenvector Dynamics Analysis (LEiDA) method, for instance, analyzes the phase synchronization of BOLD signals across different brain regions at each repetition time (TR) to effectively identify the repertoire of large-scale functional networks in different brain states [20,21]. By quantifying changes in brain states using specific metrics, such as the probability of occurrence and transition trajectories, this method allows for a more precise characterization of the dynamics of brain activity. Further, if each brain region is abstracted as an excitatory neuronal pool and an inhibitory neuronal pool, the observed changes in large-scale brain network dynamics can be seen as shifts in interactions within and between these pools [22]. Healthy brain function relies on a delicate balance between excitation (E) and inhibition (I) [23,24]. Both excessive neural activity, which can cause the network to become “noisy,” and insufficient neural activity, which can lead to an overly “quiet” network, may contribute to various psychiatric disorders [25]. However, studying the modulation of the E/I ratio by LSD in humans is challenging due to the limitations of non-invasive neuroimaging techniques. One promising approach involves the use of biophysically plausible large-scale circuit models of coupled brain regions, which, by simulating BOLD signals under both LSD and placebo conditions, can reveal changes in the E/I ratio and provide insights into how LSD modulates large-scale brain network dynamics, thereby elucidating the underlying mechanisms of its impact [26].

**Fig 1:**
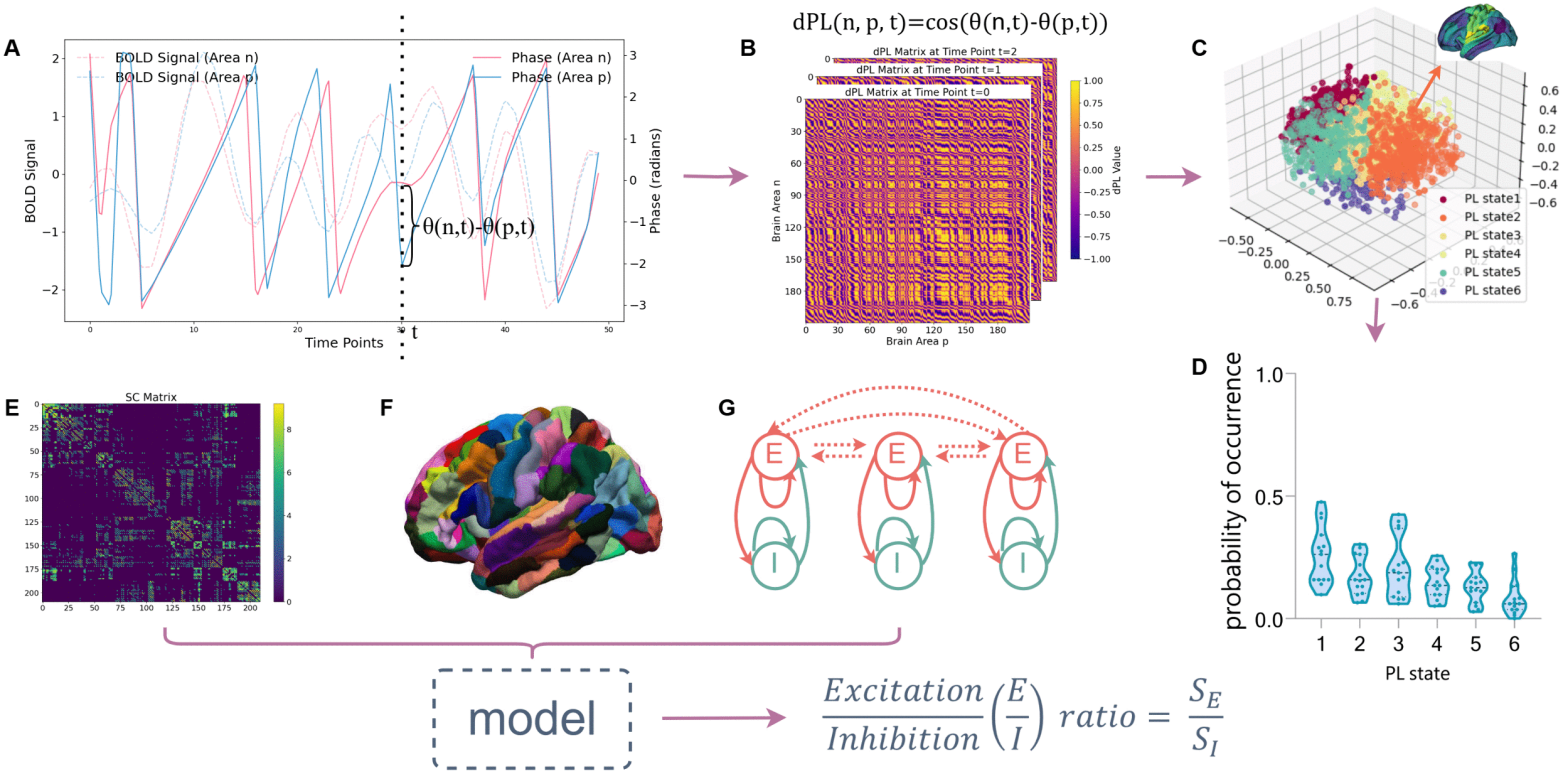
Overview of the workflow. **(A)** The phase difference estimated from the BOLD signals of brain regions n and p at time point t, obtained by applying the Hilbert transform to the BOLD signals. The dashed lines represent the BOLD signals, while the solid lines indicate the corresponding phases, with both line types using the same color for each brain region. **(B)** The phase-lock matrix calculated for a subject at t= 0, t= 1, and t= 2. **(C)** The 6 cluster centroids obtained from k-means clustering of the leading eigenvectors of the phase-locking matrices across all time points for all subjects under both LSD and placebo conditions, reduced to a 3-dimensional space, where each cluster centroid corresponds to a brain state. **(D)** Probabilities of occurrence of each brain state. **(E)** The average structural connectivity matrix. **(F)** Brainnetome Atlas (https://atlas.brainnetome.org/download.html) used for delineating brain regions; **(G)** Schematic of the neural mass model, which consists of differential equations governing the dynamics of excitatory (E) and inhibitory (I) neuron populations for each brain region. The structural connectivity, brain atlas, and dynamical model collectively determine the digital twin brain model, with the ratio of the time-averaged values of excitatory and inhibitory synaptic gating variables ($S_{E}/S_{I}$) representing the E/I ratio.

This study used the LEiDA method to characterize the LSD-induced repertoire of large-scale functional networks and the parametric feedback inhibition control (pFIC) model to derive potential markers of the E/I balance. The overall research process is shown in Fig 1. Findings indicate that LSD markedly enhances global brain synchrony by modulating the E/I ratio within specific networks, potentially driven by the Sensorimotor (SOM) cortices, whereby LSD reduces activity in SOM while facilitating enhanced engagement of the transmodal association cortex. These changes lead to a disorganization of cognitive processing, shifting the brain from a high-level cognitive control state to a non-modular, de-differentiated state, which may underlie the hallucinatory and introspective experiences induced by LSD.

## Results

Following quality control, five participants were excluded: four due to excessive head motion and one due to intra-scanner anxiety. The final sample comprised 15 participants (4 females, 11 males; mean age 30.5 ± 8.0 years) who were included in subsequent analyses.

### Global synchronization enhanced by LSD

**Fig 2:**
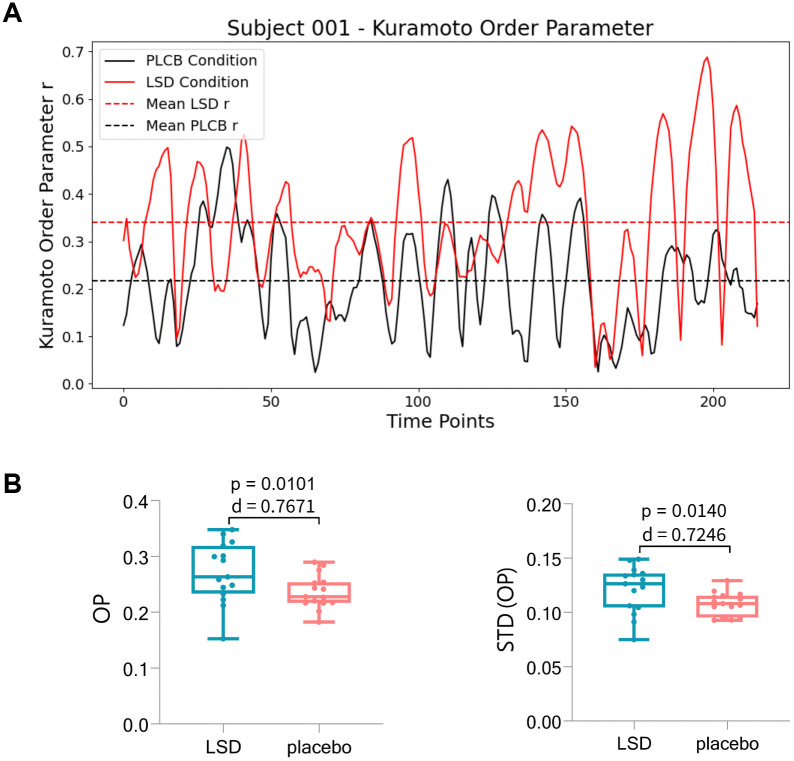
Visualization of BOLD signals and Kuramoto order parameter for subject 001 and results of paired t-test. **(A)** The Kuramoto order parameter (OP) of Subject 001 under LSD (red solid line) and placebo (black solid line) conditions across 216 time points. The dashed lines represent the mean OP over time for each condition, with a red dashed line indicating the LSD condition and a black dashed line indicating the placebo condition. **(B)** Boxplots of OP (left panel) and the standard deviation of the OP, STD(OP), (right panel) under LSD and placebo conditions. Each boxplot shows the distribution of data, with the central horizontal line representing the median, the box indicating the interquartile range (IQR) between the first (Q1) and third (Q3) quartiles, and the whiskers extending to the minimum and maximum values within 1.5 times the interquartile range from Q1 and Q3. Statistical details: OP (LSD vs. placebo), p = 0.0101, Cohen’s d = 0.7671; STD(OP) (LSD vs. placebo), p = 0.0140, Cohen’s d = 0.7246.

In this study, Kuramoto Order Parameter (OP) was employed to assess the alignment of BOLD signal phases across the entire brain under LSD and placebo conditions, with the aim of determining the impact of LSD on overall brain synchrony. Results from paired-sample t-tests indicated that the OP during the LSD condition (0.2708 ± 0.0540) was significantly higher than that observed during the placebo condition (0.2367 ± 0.0305) as shown in Fig 2, with a p-value of 0.0101 (threshold set at 0.05), and the effect size (Cohen’s d) was 0.7671. This finding suggests that LSD significantly enhances global brain synchrony, aligning with previous research [4].

Additionally, the standard deviation of the OP, STD(OP), was analyzed to evaluate the dynamic stability of the system, providing insights into the complexity of brain networks under different conditions. The results revealed that the STD(OP) in the LSD condition (0.1210 ± 0.0216) was significantly greater than that in the placebo condition (0.1079 ± 0.0106), with a p-value of 0.0140, and the effect size (Cohen’s d) was 0.7246. This indicates that under LSD, the brain network may exhibit transitions between multiple metastable states, reflecting more complex cognitive processes and richer neural activity patterns.

### Phase-Locking States (PL states) and their spatial overlap with the 7 Resting-State Networks (RSNs)

**Fig 3:**
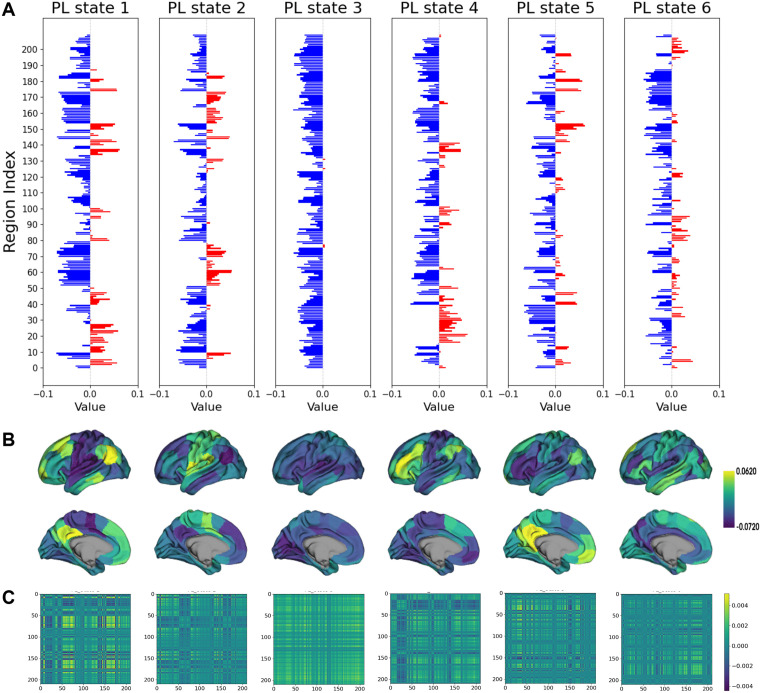
Repertoire of spatially distributed phase-locking patterns (PL states) obtained using the Leading Eigenvector Dynamics Analysis (LEiDA). Each column corresponds to a distinct brain state. **(A)** The bar plot showing the cluster centroid vectors (i.e., PL states), where each element of the centroid vector V_c_ represents the projection of the BOLD phase of each region onto V_c_. If the BOLD phase of a region aligns with the direction of V_c_, the corresponding element is positive (colored red); otherwise, it is negative (colored blue). **(B)** The representation of brain regions based on their contributions, where the magnitude of each element in V_c_ indicates the contribution of that brain region to the respective PL state, with color intensity reflecting the value. **(C)** The PL states are represented in matrix format by computing the outer product of V_c_ × V_c_^T^_,_ as depicted in the bottom panel.

To further elucidate the precise mechanisms underlying the increased synchrony of brain activity induced by LSD, LEiDA was employed. This method characterizes the phase synchronization of BOLD signals across different brain regions at each repetition time (TR). By applying k-means clustering to these synchronization patterns, we derived a repertoire of recurrent phase-locking states (PL states) across all subjects under both LSD and placebo conditions. The results for k = 6, where k represents the pre-defined number of clusters, are illustrated in Fig 3. These PL states provide a compact representation of how the brain dynamically organizes into distinct large-scale functional configurations during rest. We then quantified key dynamic properties of these states—including their probability of occurrence and dwell time—and examined the transitions between them. This approach enables a granular investigation into how LSD alters the brain’s dynamic landscape, thereby revealing the specific mechanisms behind the observed increase in synchrony. Fig 3A displays bar plots of each cluster centroid vector (i.e., PL state), where each element of the vector represents the projection of the BOLD phase of each brain region onto the leading eigenvector, indicating the contribution of that brain region to the respective PL state. Notably, PL state 3 exhibits predominantly negative values, corresponding to a global mode in which all BOLD signals change in a unified direction (i.e., all signals project towards the same direction on the leading eigenvector), consistent with findings from previous studies utilizing the LEiDA approach [20,21,27]. In addition, several non-global modes were also identified, and the transitions between these modes influenced the degree of phase alignment of the BOLD signals. The rendering of each PL state based on its values projected onto the cortical surface is depicted in Fig 3B. The matrix representation of the PL states, shown in Fig 3C, is obtained by calculating the outer product of V_c_ × V_c_^T^.

**Fig 4:**
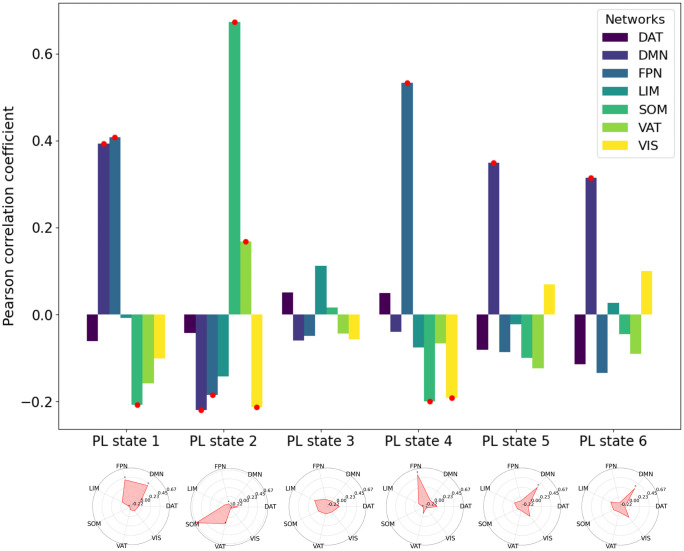
Spatial overlap of six PL states with seven resting-state networks (RSNs). The bar chart illustrates the Pearson correlation coefficients between the six PL states and the seven RSNs [28]. The length of each bar represents the magnitude of the Pearson correlation coefficient. Red circles indicate significant correlations with p-values less than 0.05 (FDR corrected). The radar plots further depict the relationships, with red dots marking the points where the p-values are less than 0.05, highlighting the significant overlaps between the PL states and RSNs in spatial representation. VIS, Visual; SOM, Somatomotor; DAT, Dorsal Attention; VAT, Ventral Attention; LIM, Limbic; FPN, Frontoparietal; DMN, Default.

To further explore the mechanism of LSD’s effects, we examined the spatial correspondence between the six extracted PL states and the seven canonical resting-state networks (RSNs) defined by Yeo et al. [28]. Specifically, we computed Pearson correlation coefficients between the spatial pattern of each PL state (represented by the cluster centroid vector V_c_, where negative elements were set to zero) and the spatial distribution of each RSN (represented by a 210-element vector encoding the contribution of each Brainnetome area to the network). This analysis quantifies the degree of spatial overlap between the functional architecture captured by each PL state and the established RSNs, as illustrated in Fig 4.

The spatial distribution of PL state 1 showed significant positive correlations with the spatial distributions of the Default Mode Network (DMN) and the Frontoparietal Network (FPN), both of which are transmodal association networks. Conversely, PL state 1 exhibited significant negative spatial correlations with the Somatomotor (SOM) and Ventral Attention (VAT) networks, which represent unimodal sensorimotor regions. Similarly, the spatial pattern of PL state 2 demonstrated significant negative correlations with the DMN and FPN networks—networks positioned at one extreme of the principal gradient—while showing positive spatial correlations with the SOM and VAT networks located at the opposite extreme. PL state 3 displayed low correlation coefficients (all < 0.12) with all RSNs, and none reached statistical significance, consistent with its globally synchronized yet functionally non-modular pattern as indicated by the nearly uniform negative values in its centroid vector. PL state 4 was significantly positively correlated with the FPN and negatively correlated with the SOM and Visual (VIS) networks in spatial distribution. Finally, both PL state 5 and PL state 6 showed significant positive spatial correlations exclusively with the DMN. All correlation coefficients and corresponding FDR corrected p-values are provided in S1 Table.

### LSD-induced transition dynamics of PL states

**Fig 5:**
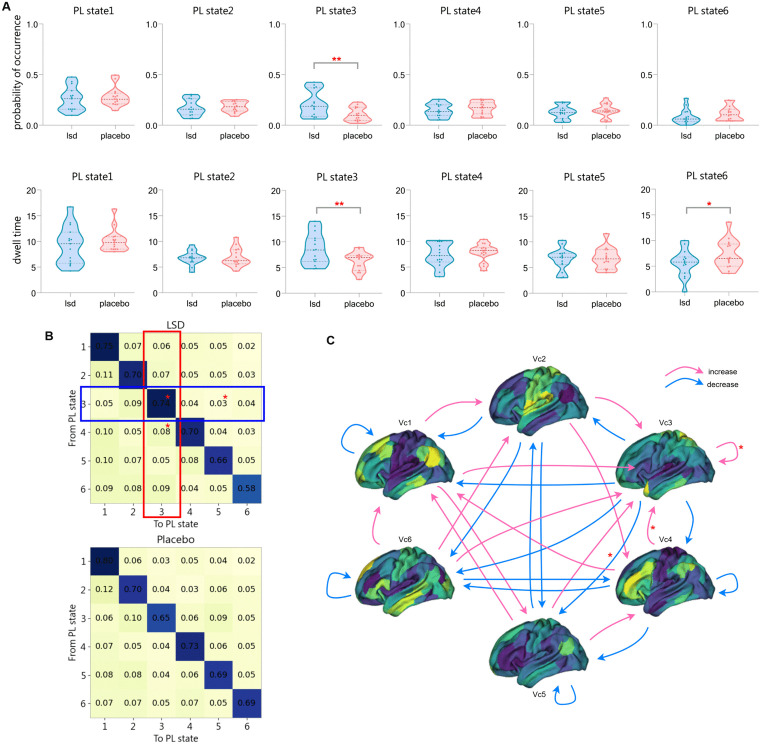
Probability of occurrence, dwell time, and transition probabilities between 6 PL states. **(A)** The first row shows the transition probabilities of the 6 PL states under LSD and placebo conditions, while the second row displays the dwell time. PL states marked with asterisks indicate significant differences assessed by permutation tests, with one asterisk representing FDR uncorrected p < 0.05 and two asterisks representing p < 0.05/k, where k = 6. **(B)** The matrix of transition probability for PL states under LSD and placebo conditions. Compared to the placebo condition, all probabilities of transitioning out of PL state 3 decreased under LSD (highlighted in the blue box), while all probabilities of transitioning into PL state 3 increased (highlighted in the red box), with significant changes indicated by asterisks. **(C)** Changes in transition probabilities between PL states under LSD compared to the placebo condition. Red/blue arrows indicate higher/lower transition probabilities in the LSD condition compared to the placebo condition, with significant differences marked by asterisks.

The paired-sample t-test, after applying FDR correction for multiple comparisons, revealed that the probability of occurrence in PL state 3 under the LSD condition was significantly higher than that under the placebo condition (p = 0.0168, Cohen’s d = 0.8661), as shown in the first row of Fig 5A. No significant group differences were found for the other PL states (see S2 Table for detailed p-values and effect sizes).

In terms of dwell time, after FDR correction, only PL state 3 showed a significant between-group difference (p = 0.0264, Cohen’s d = 0.8386). Additionally, although not surviving FDR correction, PL state 6 exhibited a trend towards shorter dwell time under the LSD condition compared to the placebo condition (uncorrected p = 0.0044, Cohen’s d = -0.6657), as shown in the second row of Fig 5A. No significant group differences were found for the other PL states (see S3 Table for FDR corrected p-values and effect sizes).

Considering the increased global brain activity synchronization mentioned earlier, it can be speculated that the increase in the probability of occurrence of PL state 3, which represents a global connectivity pattern, under the LSD condition may contribute to the overall higher synchronization of brain activity. After applying FDR correction, the correlation between the probability of occurrence of the different PL states and OP revealed a highly significant positive correlation between PL state 3 and OP (r = 0.8632, p < 0.0001), which supports this hypothesis. The probability of occurrence for both PL state 2 and PL state 6 was significantly negatively correlated with OP, with correlation coefficients of r = -0.4997 (p = 0.0147) and r = -0.4796 (p = 0.0147), respectively. Correlation results for the remaining PL states can be found in S4 Table. Furthermore, significant correlations were found between the probability of occurrence of PL state 3, and PL state 6 and STD(OP). These changes in the probability of occurrence of the three PL states also contribute to the increased dynamic flexibility of brain activity.

To better understand the dynamic trajectories of the PL states, Fig 5B presents the probability matrices for transitions between a given PL state (rows) and other PL states (columns) under LSD (upper panel) and placebo conditions (lower panel). The results show that, compared to the placebo condition, the probabilities of transitioning from PL state 3 to other PL states were all reduced under LSD. Notably, a nominal significance (i.e., uncorrected p < 0.05) was observed for the difference in the probability of transitioning from PL state 3 to itself (uncorrected p = 0.0338, Cohen’s d = 0.7273), and for the transition to PL state 5 (uncorrected p = 0.0066, Cohen’s d = -0.6535). In contrast, the probabilities of transitioning from other PL states to PL state 3 were all increased, with the transition from PL state 4 to PL state 3 also showing a nominally significant difference (uncorrected p = 0.0376, Cohen’s d = 0.5201). However, it is important to note that after applying False Discovery Rate (FDR) correction for multiple comparisons across all state transitions, none of these differences remained statistically significant (all pFDR > 0.05). Despite this, the consistent direction of effects across related transitions and the moderate effect sizes suggest that these patterns may hold biological relevance and warrant further investigation in larger studies. To enhance clarity, Fig 5C displays this result with arrows in different colors. Results of the other paired-sample t-tests can be found in S5 Table.

### Consistency of results across different k-values

**Fig 6:**
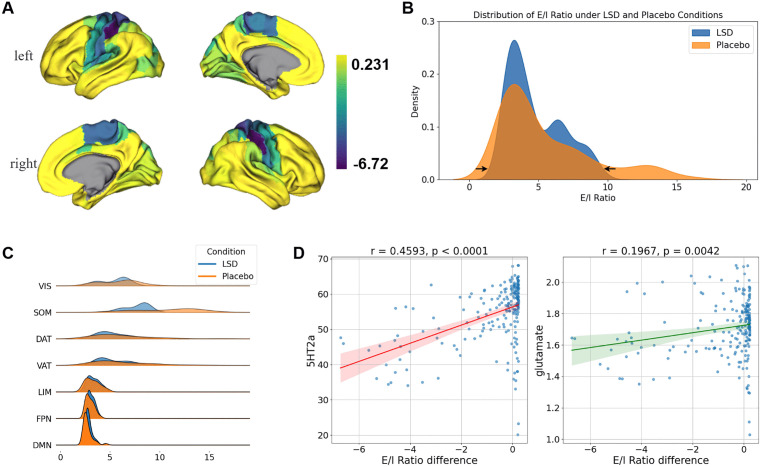
Distribution of E/I Ratio Differences on the Cortex. **(A)** The distribution of the E/I ratio rendered onto the cortical surface. **(B)** The administration of LSD induces a compression of the global E/I ratio towards the center along the principal functional gradient. **(C)** The distribution of E/I ratios across the 7 RSNs under LSD and placebo conditions shows that the peak E/I ratio in the primary cortex under LSD is situated to the left of the corresponding peak observed under placebo, whereas the peak in the associative cortex under LSD is located to the right of the placebo peak. **(D)** Pearson correlation between the E/I ratio difference and the densities of 5-hydroxytryptamine-2A (5-HT2A) receptors and glutamate receptors.

To determine whether the results were influenced by the value of k, we identified the PL states with the most significant group differences in probability of occurrence and dwell time between LSD and placebo conditions across various partitioning models. For partitioning models with k values of 2, 3, and 4, no significant group differences were found for any PL state in terms of probability of occurrence or dwell time. However, for partitioning models with k values ranging from 5 to 10, significant group differences were observed for certain PL states (see S6 Table for detailed p-values). The PL states with the most significant differences are shown in Fig 6. The bar charts in S1A Fig clearly show that for models with k values from 5 to 10, the cluster center vectors corresponding to significant PL states at each k value are predominantly negative. These PL states also exhibit very similar cortical distribution patterns, all representing global connectivity, as shown in S1B Fig. Additionally, Pearson correlations were calculated between these states, revealing that, with the exception of the PL state with the most significant differences at k = 5, which showed the lowest correlation (r = 0.78) with the most significant PL states at other k values, all other k values showed high correlations (r ≥ 0.95) between the most significantly differing PL states, as shown in S2 Fig. This likely occurs because at a lower k-value (k = 5), the clustering algorithm merges slightly distinct “global states” that are separated at finer partitions (k > 5), leading to a centroid that differs subtly from the “purer” global state identified for k ≥ 6. Beyond this technical observation, the stable emergence of this specific “global state” across multiple partition scales (k-values) suggests it may represent a fundamental or robust dynamical attractor towards which the brain is driven under LSD.

### LSD-induced E/I ratio differences exhibit network-specific effects

Under the optimal parameters, the Pearson correlation coefficient (r) between the empirical and simulated FC for the LSD condition in the validation set was 0.34827, with a p-value less than 0.0001. For the placebo condition, the corresponding r value was 0.30049, with a p-value also less than 0.0001. The absolute difference (d) between the empirical and simulated FC was 0.0193 for the LSD condition and 0.0442 for the placebo condition. Additionally, the KS distance between the empirical and simulated FCD was 0.0482 for the LSD condition and 0.0719 for the placebo condition. These results suggest that the pFIC model generated realistic FC and FCD metrics.

Intuitively, regions with a negative E/I ratio difference in the cortical areas were primarily concentrated in SOM, as shown in Fig 6A. After FDR correction for multiple comparisons, the Pearson correlation results between the E/I ratio difference and the 7 RSNs revealed a significant negative correlation with SOM (r = -0.6286, p < 0.0001), and significant positive correlations with DMN (r = 0.2410, p = 0.0009), FPN (r = 0.2012, p = 0.0079), and LIM (r = 0.1770, p = 0.0178). The remaining correlation results are presented in S7 Table. As shown in Fig 6B, the distribution of the E/I ratio difference data for the LSD condition was more compact compared to the placebo condition, with closely aligned peak data densities for both conditions. When examining the E/I ratio differences for each of the 7 RSNs separately, the data distribution in the LSD condition was consistently more compact across all RSNs. Specifically, as shown in Fig 6C, the peak data densities for VIS, SOM, DAT, and VAT in the LSD condition were located to the left (i.e., smaller values) of those in the placebo condition, whereas the peak data densities for LIM, FPN, and DMN were located to the right (i.e., larger values) of the placebo condition distributions. The Wilcoxon signed-rank test revealed that the E/I ratio differences for all brain networks were significantly greater than zero for VIS, SOM, DAT, and VAT, or smaller than zero for LIM, FPN, and DMN. The p-value for LIM was 0.001, and all others were less than 0.001.

A key mechanism of LSD’s effects is its ability to stimulate the 5-hydroxytryptamine-2A (5-HT_2A_) receptors, which in turn activates glutamatergic transmission in the prefrontal cortex, ultimately influencing downstream subcortical regions and altering sensory and cognitive processing [29]. To further investigate this relationship, we calculated the Pearson correlations between the E/I ratio difference and the densities of 5-HT_2A_ and glutamate receptors. Results revealed significant positive correlations between the E/I ratio difference and both 5-HT_2A_ receptor density (r = 0.4593, p < 0.0001) and glutamate receptor density (r = 0.1967, p = 0.0042), as shown in Fig 6D.

## Discussion

Present study, from the perspective of phase coherence, found that LSD significantly enhances global brain synchrony and promotes a broader range of functional metastable states. By extracting the repertoire of metastable activity patterns induced by LSD, it was observed that the increased global synchrony is associated with an increased probability of the PL state 3, which is a non-modular pattern, and a decreased probability of the PL state 2 and PL state 6, which are denoted as local connectivity patterns. Notably, PL state 2 is primarily localized to the SOM cortices, while PL state 6 significantly overlaps spatially with the DMN. In this context, the modulation of the E/I ratio in specific brain networks was further explored. Estimating the E/I ratio under both LSD and placebo conditions revealed that LSD modulates the E/I ratio in a network-specific manner, with the sensorimotor systems driving these effects. Specifically, in the transmodal association cortex (e.g., DMN, FPN, and LIM), the increased E/I ratio may reflect reduced inhibition or enhanced excitation, leading to overactive neurons. Conversely, the reduced E/I ratio in the primary sensorimotor systems suggests a diminished response to external sensory stimuli, facilitating a more introspective state.

The findings of the present study regarding enhanced brain synchrony under LSD are consistent with previous research. Earlier research demonstrated that LSD enhances functional connectivity between brain regions [4]. In the current study, the OP of the BOLD signal was significantly higher under LSD than placebo. Additionally, the repertoire of metastable activity patterns revealed that PL states (1, 2, and 4) showed both positive or negative correlations with the spatial distribution of multiple resting-state networks (RSNs), suggesting that LSD facilitates the involvement of different brain regions across multiple networks and implying greater cross-network connectivity, corroborating prior findings. Furthermore, a previous study reported that LSD reduces functional segregation between brain networks [30], a finding also reflected in the current study by an increased probability of PL state 3, a non-modular pattern. The strong positive correlation between the probability of PL state 3 and OP may indicate that LSD enhances the dynamic flexibility of brain networks, reducing the rigidity of brain activity. This could facilitate rapid transitions between different brain states and adaptive changes, which might be associated with the altered states of consciousness and increased cognitive flexibility reported in previous studies of LSD. In other words, LSD enhances the connections between brain networks that are typically more segregated during resting states, effectively blurring the boundaries between previously independent brain regions. Moreover, the significant increase in the standard deviation of OP under LSD suggests that LSD induces more metastable states and enhances dynamic complexity in brain networks. This finding is consistent with the previous research, which reported an increase in brain entropy under LSD [31], indicating that LSD makes brain activity more complex, variable, and unpredictable, with a more flexible and dynamic activity pattern [32].

LSD administration reduces the transition probability from PL state 3 to other states, with a notable decrease in the probability of transitioning to PL state 5. PL state 3 does not exhibit significant overlap with any major sensory processing networks, and may represent a pattern characterized by functional decoupling from the canonical modular architecture of resting-state networks, possibly associated with fundamental self-regulation or the monitoring state of the brain. PL state 5, in contrast, shows a strong positive correlation with DMN. Previous research has described a local functional gradient within the cortical areas, ranging from sensory and motor regions to more abstract functional cortical areas [33–36]. At one end of this gradient lies the primary sensorimotor regions, while the other end is connected to transmodal regions, which are known as the DMN in humans, responsible for processing transmodal information independent of sensory input [37–40]. Mesulam’s work suggested that more abstract functional classes may follow a similar pathway, with these abstract categories emerging through the integration of transmodal information [41]. Therefore, the reduced transition probability from PL state 3 to PL state 5 under LSD reflects a suppression of the transition from standby state to a more highly integrated cognitive state, indicating a potential reorganization of brain activity under LSD. Conversely, the transition probability from other states to PL state 3 increases across the board, especially for the transition from PL state 4 to PL state 3, which is enhanced under LSD conditions. PL state 4, in spatial distribution, is significantly positively correlated with FPN and negatively correlated with both the SOM and VIS networks. The “Tethering Hypothesis” [42] posits that the functional properties of association cortices arise because they are progressively less constrained by the spatial boundaries that define primary cortical areas, such as sensory and motor cortices. Thus, PL state 4 likely represents a state involving higher cognitive functions, attention regulation, and cognitive control, where the influences of sensory input and motor activity are suppressed or decoupled, with a greater focus on cognitive control and introspective processes. Under LSD conditions, the significantly increased transition probability from PL state 4 to PL state 3 may indicate that LSD suppresses cognitive control networks (e.g., FPN) [43,44], thereby shifting the brain from a higher cognitive control state (PL state 4) to a more free or sensitive standby state (PL state 3).

Previous studies suggest that cognitive disorganization may be a more fundamental characteristic of the effects of LSD, rather than simply positive or negative emotional states [29,45]. Additionally, the influence of the E/I ratio on cognitive abilities varies across different cortical regions, with the associative cortices showing the most significant impact and the sensorimotor cortices the least, indicating a closer relationship between the functional properties of the associative cortex and cognitive ability [26,46,47]. The present study supports these conclusions by demonstrating an uneven E/I ratio change along the principal functional gradient induced by LSD. Specifically, we found that LSD increased the E/I ratio in the associative systems (positive deviation), suggesting a “reset” or enhanced plasticity state in the associative system, where neurons become hyperactive in processing information, thus fostering cognitive flexibility and creativity. However, this does not equate to improved cognitive ability, as a network composed solely of excitatory neurons would have reduced computational complexity and limited information-processing capacity [48]. In other words, purely excitatory connections are incapable of handling complex information processing [49,50]; the presence of GABAergic inhibitory interneurons acts as a “brake” to control the transmission of excitatory signals, preventing excessive feedforward excitation from afferent structures [51, 52]. The reduction in the E/I ratio in the primary sensorimotor system may lead to a diminished response to external sensory stimuli, potentially affecting sensory information processing. This inhibitory effect could facilitate an introspective state during the LSD experience, reducing focus on external stimuli. This aligns with previous conclusions suggesting that LSD seems to alter spontaneous perception rather than directly induce sensory responses [53]. Furthermore, the E/I ratio difference was primarily distributed within the sensorimotor cortices. It has been demonstrated that the spontaneous amplitude fluctuations of sensory-motor regions are a driver of the switching between a high coherent state and a low coherent state [54]. Notably, the probability of occurrence of PL state 2, which spatially overlaps primarily with SOM, showed a significant negative correlation with OP. This finding can be interpreted in the context of Dong et al. (2023), who suggested that disturbances in early regions of sensory pathways may lead to bottom-up dysregulation of higher-order cortical functions [55]. The convergence of these experimental and theoretical lines of evidence suggests that LSD-induced alterations in sensory processing networks might constitute a primary driver of the observed global synchrony changes, though direct causal evidence remains to be established in future studies. Overall, the E/I ratio differences demonstrated a compression along the principal gradient of the cortex, with de-differentiation of unimodal and transmodal cortices, which has been reported in prior studies [3]. Specifically, serotonergic psychedelics have been shown to reduce intermediary processing steps between unimodal and transmodal cortices, diminishing the functional differentiation between sensory processing and abstract cognitive processing, while increasing global connectivity within transmodal cortices, which disrupts the specialized processing of unimodal sensory information by enhancing overall cortical synchrony [30,56–58]. This mechanism may be explained by LSD and other stimulation of 5-HT2A receptors by serotonergic psychedelics [59,60], which activate glutamatergic transmission in the prefrontal cortex [61,62]. Our findings support this by showing positive correlations between the E/I ratio difference and the densities of both 5-HT2A and glutamate receptors.

Overall, the effects of LSD on the cortical network observed here may facilitate the emergence of de-differentiated brain activity patterns. Speculatively, such patterns could enhance flexibility and creativity, while potentially limiting the depth of information processing. The dual nature of these effects highlights their intriguing, yet complex, potential relevance for mental health applications, which merits further investigation that includes psychometric correlates. Notably, SOM may serve as a key driving region underlying these effects. These results advance our understanding of how LSD modulates neural systems, providing a framework for future investigations into both the neurobiological mechanisms of psychedelics and their potential clinical applications.

This study has several limitations that should be acknowledged. First, although the LEiDA method, which characterizes brain network dynamics at each time point, helps mitigate the limitations associated with the small sample size, the division of 15 participants into training and validation sets still results in a limited dataset. Larger sample sizes would undoubtedly lead to more reliable and generalizable results. Second, this study primarily focused on the overall synchrony of brain activity and the coordination and specificity of large-scale resting-state networks under LSD, without delving into the relationship between these effects and specific dimensions of psychometric scales. Additionally, this study focused on large-scale cortical networks, and consequently, the role of subcortical structures (e.g., the thalamus) was not incorporated into our models. Given the established importance of these regions in psychedelic action, their inclusion in future work is crucial to bridge our system-level findings with the specific subcortical regions that may orchestrate these dynamics.

## Materials and methods

### Ethics statement

The experimental protocol received approval from the West London Research Ethics Committee of the UK National Health Service. The experiments adhered to the revised Declaration of Helsinki (2000), the International Council for Harmonisation Good Clinical Practice guidelines, and the National Health Service Research Governance Framework. Twenty participants were recruited via word of mouth and provided written informed consent after a thorough study briefing and screening for physical and mental health.

### Functional magnetic resonance imaging (fMRI) data

The present analysis utilized neuroimaging data from a publicly available dataset on OpenNeuro, with detailed information provided in the original publication [4]. The screening included an electrocardiogram (ECG), routine blood tests, and a urine test to check for recent drug use and pregnancy. Participants also underwent a psychiatric interview to disclose their complete drug use history. Key exclusion criteria included being under 21 years of age, personal history of diagnosed psychiatric illness, immediate family history of psychotic disorders, lack of prior experience with classic psychedelic drugs (such as LSD, mescaline, psilocybin, or DMT), any psychedelic drug use within six weeks prior to the first scanning day, pregnancy, problematic alcohol consumption (more than 40 units per week), or any medically significant conditions that would render the volunteer unsuitable for the study.

Following the initial exclusion criteria, 20 healthy participants underwent resting-state fMRI Blood Oxygen Level Dependent (BOLD) data acquisition under both LSD and placebo conditions, with a minimum interval of 14 days between the two conditions. The order of administration of LSD or placebo was counterbalanced across subjects to control for potential order effects. Participants were blinded to the order of administration, while the researchers were informed of it. On each scanning day, each participant received an intravenous injection of 75 µg of LSD dissolved in 10 mL of saline for the LSD condition, or 10 mL of saline for the placebo condition, 70 minutes prior to the MRI scan. The acquisition protocol is elaborated upon in greater detail in the original publication [4]. The fMRI BOLD data were recorded using a specific gradient echo planar imaging sequence and underwent detailed preprocessing steps as described in the original publication [31].

### fMRI processing

BRANT version 3.35, a MATLAB toolbox specifically designed for the batch preprocessing of fMRI data, was utilized in this study [63]. The preprocessing procedures were comprehensively detailed by [64]. Following a series of preprocessing steps, including band-pass filtering (0.01–0.08 Hz) and motion correction, BOLD time series were extracted for each brain region. Specifically, for each participant at each condition, the fMRI data were averaged within the 210 regions of interest (ROIs) defined by the Brainnetome Atlas, resulting in a 210 (ROIs) × 216 (time points) matrix.

These matrices were then used to compute functional connectivity (FC) matrices (210 × 210) by calculating the pairwise correlations between the time courses of all ROIs. The group-level FC matrix was computed by averaging the participant-level FC matrices separately for the training (n = 7) and validation (n = 8) sets, with 15 participants randomly assigned to each set. The calculation of the Functional Connectivity Dynamics (FCD) matrix was carried out as follows. A sliding window of 60 seconds (equivalent to 30 TRs, given a TR of 2 seconds) was employed, consistent with recommendations from prior research [26]. This window was shifted from the first frame to the 187th frame of the BOLD time series, resulting in a total of 187 sliding windows. Each FC matrix corresponding to a sliding window was then vectorized by focusing solely on the upper triangular entries, and the vectorized FCs were subsequently correlated with one another, generating a 187 × 187 FCD matrix.

### Structural connectivity

The diffusion tensor imaging (DTI) data from 1065 participants in the Human Connectome Project (HCP) S1200 release were utilized in this study. Detailed information regarding data collection is available in previous literature [65]. The participants were randomly divided into a training set (N = 532) and a validation set (N = 533).

To construct structural connectivity (SC) matrices for each participant, whole-brain tractography was performed using the fiber orientation distribution (iFOD2) algorithm [66] provided by MRtrix3 [67]. Each SC matrix entry represented the number of streamlines between two regions of interest (ROIs). A thresholding procedure was applied to eliminate false positives, setting entries to zero if fewer than 50% of participants exhibited a non-zero value. The number of streamlines was averaged across participants with non-zero values and log-transformed [67], while the main diagonal entries were set to zero. Group-level SC matrices were generated by averaging the individual-level SCs for both the training and validation sets, with the maximum value normalized to 0.02 following the previous study [26].

### Global synchrony and metastability assessment

To evaluate the synchrony and metastability of brain network dynamics, we utilized the Kuramoto order parameter (OP), a metric commonly employed to quantify the collective dynamics of coupled oscillators, especially in systems consisting of multiple interacting units [68–71]. This parameter provides data-driven framework for measuring the extent of phase coherence or the degree of synchrony among the oscillators, here, the brain regions, wherein the phase represents a large-scale hemodynamic rhythm rather than the instantaneous phase of neuronal spiking. Specifically, the Kuramoto order parameter at time point *t* is defined as:


$$\begin{array}{c} OP(t)=\frac{\text{1}}{N}|\sum_{i=\text{1}}^{N}e^{i\theta (n,\;t)}| \end{array}$$ (1)

where *N* is the number of oscillators, corresponding to the 210 brain regions in this study, and θ(n, t) represents the phase of the region *n* at time point *t*.

In the study, the BOLD signal from brain region *n* was treated as a continuous oscillatory signal x(n, t). The Hilbert transform takes this real-valued signal x(n, t) and creates a 90-degree phase-shifted version $\hat{x}(n,\;t)$. Then the original signal x(n, t) and its Hilbert transform $\hat{x}(n,\;t)$ were combined to form the analytic signal $z(n,\;t)=x(n,\;t)+i\hat{x}(n,\;t)$, which can be expressed as $A(n,\;t)e^{i\theta (n,\;\;t)}$, where A(n, t) is the instantaneous amplitude and θ(n, t) is the instantaneous phase [20,72]. The phase θ(t) of each brain region was then used to compute the Kuramoto order parameter at each time point.

The mean OP across time provides a measure of the system’s overall synchrony. A value close to 0 indicates that the system remains largely desynchronized, with the brain regions operating independently and showing minimal phase coherence. A value between 0 and 1 suggests partial synchrony, where certain periods exhibit some degree of coordination among brain regions. A value approaching 1 indicates full synchrony, with most brain regions oscillating in phase, reflecting coordinated network activity. The standard deviation of the OP, STD(OP), was used to quantify the metastability of the system [73,74]. The STD(OP) reflects the temporal variability of synchrony: when OP remains relatively stable over time, it suggests that the system is in a stable equilibrium, whether synchronized or desynchronized. On the other hand, a higher standard deviation indicates that the system is dynamically shifting between different states of synchrony, characteristic of a metastable system, and implies that the system is exploring a broader range of functional metastable states. Finally, a paired-samples t-test was conducted to evaluate the differences in the OP and STD(OP) between the LSD and placebo conditions.

### Leading Eigenvector Dynamics Analysis (LEiDA)

To extract the repertoire of large-scale functional networks, the phase-locking matrix was computed for each subject across 216 time points:


$$\begin{array}{c} dPL(n,p,t)=\text{cos}\left(\theta (n,t)-\theta (p,t)\right) \end{array}$$ (2)

where θ(n,t) and θ(p,t) denote the instantaneous phase of brain region *n* and *p*, as shown in Fig 1A. This resulted in a three-dimensional matrix of size N × N × T for each subject, where N = 210 as shown in Fig 1B. Then, the leading eigenvector *V*_*1*_*(t)* of the phase-locking matrix for each subject at each time point *t* was computed, which is an N × 1 vector serving as a low-dimensional representation of the BOLD phase-locking patterns, encapsula*t*ing the primary orientation of BOLD phases across all brain areas. Each element of *V*_*1*_*(t)* reflects the projection of the BOLD phase of a given brain area into this leading eigenvector, indicating the degree to which each area aligns with the overall network mode. When all elements of *V*_*1*_*(t)* share the same sign (either all positive or all negative), it indicates a state of full synchrony, where all brain regions oscillate in phase with respect to *V*_*1*_*(t)*. Conversely, differing signs among the elements suggest the presence of distinct clusters of brain areas, each exhibiting different phase relationships with respect to the leading eigenvector *V*_*1*_*(t)*.

To identify recurrent phase-locking states, k-means clustering was applied to the collection of leading eigenvectors *V*_*1*_*(t)* across all subjects, time points, and conditions. A range of cluster numbers *k* from 2 to 10 was explored, allowing for the capture of varying levels of functional network granularity. In the current analysis, we identified the cluster solution k = 6 as the most favorable option by considering four evaluation metrics—Dunn score, distortion, silhouette score, and Davies–Bouldin score—while also aiming to optimize the statistical significance of the differences observed between patient groups. The resulting cluster centroids, denoted as V_c_, represent distinct phase-locking states (PL states), capturing recurrent patterns of phase synchrony across the brain, which can be interpreted as metastable brain network configurations. Each PL state can be visualized as a network in cortical space, as shown in Fig 1C, where the magnitude of *V*_*1*_*(t)* (the *n-th* element of the cluster center vector) is used to scale the color of each brain area, highlighting the relative contributions of different regions to the network.

Given that both V and -V occupy the same one-dimensional subspace, we adopted a convention where the majority of elements are negative. This choice is supported by previous study [27], which indicates that the smallest subset of regions, whose BOLD phases diverge from the global mode, corresponds to significant functional brain networks, specifically the canonical resting-state networks.

### Spatial overlap with 7 Resting-State Networks

In this study, Pearson correlation was employed to assess the spatial overlap between the PL states and the seven resting state networks (RSNs) [27]. Initially, the seven RSNs defined by Yeo et al. (2011) [28] were transformed from the 1 mm³ MNI space into the Brainnetome Atlas space. This was achieved by counting the number of 1 mm³ MNI voxels within each Brainnetome area that belong to each of the seven networks, resulting in a vector with 210 elements that reflects the contribution of each Brainnetome area to the corresponding RSN. Subsequently, the Pearson correlation coefficients were computed between each pair of the 6 PL states and the seven 210-element vectors. The resulting p-values were corrected for multiple comparisons using the false discovery rate (FDR) procedure (q < 0.05). During this process, all negative elements in the V_c_ vector were set to zero, retaining only the positive values [27].

### Analysis of PL state occurrence and transitions

The clustering algorithm assigns each TR to the closest centroid $V_{C}$, thereby designating a specific PL state for each time point of each subject, facilitating the calculation of the probability of occurrence for each PL state, dwell time, as well as the state transition probability matrices. The probability of occurrence for each PL state was computed by dividing the number of epochs assigned to that PL state by the total number of epochs within each scanning session for each partition model (ranging from k = 2 to k = 10), as shown in Fig 1D for k = 6. Additionally, the dwell time was obtained by calculating the average duration spent continuously in a specific PL state throughout the entire scan.

To assess the differences in probabilities of occurrence between LSD and placebo conditions, a permutation-based paired t-test was employed. This non-parametric test generates the null distribution by randomly permuting the group labels (LSD and placebo) for each subject. For each of 1000 permutations, a paired t-test was applied to compare the populations, resulting in corresponding p-values.

To further investigate the dynamics between different PL states, switching probability matrices were constructed to represent the mean probabilities of being in a given PL state (rows) and transitioning to other PL states (columns) under both LSD and placebo conditions. The switching probabilities were determined for each subject under both conditions, and inter-group differences were statistically evaluated using a permutation-based paired t-test, with 1000 permutations.

### Simulation of E/I Ratio using the Parametric Feedback Inhibition Control (pFIC) model

The large-scale spontaneous brain activity is believed to arise from the interactions of intrinsic dynamics within local circuits over long-range anatomical connections [54,75,76]. To understand the mechanisms by which LSD affects brain networks from a neurobiological perspective, we simulated the BOLD signals and estimated E/I ratio distribution under both LSD and placebo conditions. Previous studies have demonstrated that parameterizing local circuit properties using anatomical and functional gradients can yield more realistic static and dynamic resting-state FC [26,54]. This heterogeneity of local circuits can be informed by in vivo structural and functional neuroimaging measurements. The Parametric Feedback Inhibition Control (pFIC) Model incorporates estimates of cortical myelin derived from T1-weighted/T2-weighted (T1w/T2w) MRI and the principal resting-state FC gradient, both of which have been shown to reflect anatomical and functional hierarchies [77,78].

The derivation of the FIC model has been extensively detailed in a prior study [79]. In this section, we aim to offer some insights into the FIC model. The neuronal dynamics of the *j*-th cortical region are governed by the nonlinear differential equations presented below:


$$\begin{array}{c} I_{j}^{\left(E\right)}=W_{E}I_{\text{0}}+\omega_{EE}J_{NMDA}S_{j}^{\left(E\right)}+GJ_{NMDA}\sum_{k}C_{jk}S_{k}^{\left(E\right)}-\omega_{IE}S_{j}^{\left(I\right)} \end{array}$$ (3)

$$\begin{array}{c} I_{j}^{\left(I\right)}=W_{I}I_{\text{0}}+\omega_{EI}J_{NMDA}S_{j}^{\left(E\right)}-\omega_{II}S_{j}^{\left(I\right)} \end{array}$$ (4)

$$\begin{array}{c} r_{j}^{\left(E\right)}=\phi \left(I_{j}^{\left(E\right)}\right)=\frac{a_{E}I_{j}^{\left(E\right)}-b_{E}}{\text{1}-exp\left(-d_{E}\left(a_{E}I_{j}^{\left(E\right)}-b_{E}\right)\right)} \end{array}$$ (5)

$$\begin{array}{c} r_{j}^{\left(I\right)}=\phi \left(I_{j}^{\left(I\right)}\right)=\frac{a_{I}I_{j}^{\left(I\right)}-b_{I}}{\text{1}-exp\left(-d_{I}\left(a_{I}I_{j}^{\left(I\right)}-b_{I}\right)\right)} \end{array}$$ (6)

$$\begin{array}{c} \frac{dS_{j}^{\left(E\right)}}{dt}=-\frac{S_{j}^{\left(E\right)}}{\tau_{E}}+\left(\text{1}-S_{j}^{\left(E\right)}\right)\gamma r_{j}^{\left(E\right)}+\sigma \upsilon_{j}(t) \end{array}$$ (7)

$$\begin{array}{c} \frac{dS_{j}^{\left(I\right)}}{dt}=-\frac{S_{j}^{\left(I\right)}}{\tau_{I}}+r_{j}^{\left(I\right)}+\sigma \upsilon_{j}(t) \end{array}$$ (8)

where *I*_*j*_, *r*_*j*_, and *S*_*j*_ represent the synaptic currents, firing rates, and synaptic gating variables of the excitatory (E) or inhibitory (I) neuronal populations in region *j*, respectively [22]. The first component of *I*_*j*_, denoted as $WI_{\text{0}}$, represents the overall effective external input, where *W*_*E*_ = 1, *W*_*I*_ = 0.7, and *I*_*0*_ = 0.382nA. The second component accounts for the intra-regional E-to-E currents, with *ω*_*EE*_ and *ω*_*EI*_ representing the weights of recurrent excitation and excitatory-to-inhibitory connections, respectively. The third component captures inter-regional inputs, where *G* is a global coupling factor which are unknown parameters to be estimated by fitting to empirical fMRI data and uniformly scales all E-to-E connections across regions, as determined by the structural connectivity (SC) matrix, represented in *C*_*jk*_ (the connection between region *j* and *k*). The fourth component describes the local negative feedback from the inhibitory population, governed by the strength of the inhibitory-to-excitatory connection *ω*_*IE*_ (calculated analytically to maintain a typical noisy spontaneous activity with low firing rate of 3Hz observed in electrophysiology experiments to emulate resting-state conditions) [79–81] and the strength of the inhibitory-to-inhibitory recurrent connection *ω*_*II*_ (set to 1).

*ϕ* denotes the neuronal response function [82] that converts the input current *I*_*j*_ into the firing rate *r*_*j*_. The parameter *a* serves as the gain factor that determines the slope of *ϕ*, with *a*_*E*_ = 310n/C and *a*_*I*_ = 615n/C. *b* represents the threshold current, above which the firing rate *r*_*j*_ increases linearly with the input current, where *b*_*E*_ = 125Hz and *b*_*I*_ = 177Hz. *d* is a constant that determines the curvature of *ϕ* around the threshold current, with *d*_*E*_ = 0.16s and *d*_*I*_ = 0.087s.

The synaptic gating variable *S*_*j*_^*(E)*^ of the excitatory pool is regulated by N-methyl-D-aspartate (NMDA) receptors with a decay time constant of *τ*_*E*_ = 0.1s and *γ* = 0.641. In contrast, the synaptic gating variable *S*_*j*_^*(I)*^ of the inhibitory pool primarily depends on γ-aminobutyric acid (GABA), with a decay time constant of *τ*_*I*_ = 0.01s. Additionally, *v*_*j*_ represents an uncorrelated standard Gaussian noise with an amplitude of *σ* = 0.01nA. All parameter settings were based on previous studies [83]. In this study, the regional E/I ratio is defined as the ratio of the temporal average of the excitatory synaptic gating variable *S*_*j*_^*(E)*^ to the inhibitory synaptic gating variable *S*_*j*_^*(I)*^ of this region. Using the simulated excitatory synaptic gating variables *S*_*j*_^*(E)*^ as inputs for the Balloon-Windkessel hemodynamic model [79,84,85] to simulate fMRI BOLD signals allows for the subsequent generation of simulated static FC and FCD.

As mentioned earlier, spatial heterogeneity in the excitatory-to-excitatory recurrent connection strength *ω*_*EE*_, excitatory-to-inhibitory connection strength *ω*_*EI*_, and regional noise amplitude *σ* must be taken into account in order to simulate more realistic brain activity. The use of Brainnetome Atlas with 210 cortical regions results in 210 × 3 + 1 (where the additional 1 represents the global coupling constant *G*) unknown parameters, posing a significant computational challenge. In this study, the pFIC model integrates estimates of cortical myelin derived from T1w/T2w MRI and the principal resting-state functional connectivity (FC) gradient by parameterizing *ω*_*EE*_, *ω*_*EI*_, and *σ* as a linear combination of the myelin map and the FC gradient, reducing the number of free parameters to 3 × 3 + 1 while still simulating more realistic brain activity, as follows:


$$\begin{array}{c} \omega_{EE,j}=a_{\text{1}}+b_{\text{1}}\times {myelin}_{j}+c_{\text{1}}\times FC\;{gradient}_{j} \end{array}$$ (9)

$$\begin{array}{c} \omega_{EI,j}=a_{\text{2}}+b_{\text{2}}\times {myelin}_{j}+c_{\text{2}}\times FC\;{gradient}_{j} \end{array}$$ (10)

$$\begin{array}{c} \sigma_{j}=a_{\text{3}}+b_{\text{3}}\times {myelin}_{j}+c_{\text{3}}\times FC\;{gradient}_{j} \end{array}$$ (11)

Following previous studies [26,54], the objective of the fitting is to minimize the function (1−r)+d+KS, where *r* is the Pearson correlation coefficient between the empirical and simulated FC matrices, *d* represents the absolute difference between the average empirical FC matrices and simulated FC matrices, the inclusion of which accounts for scale differences between the empirical and simulated FC matrices. *KS* denotes the Kolmogorov-Smirnov (KS) distance. Specifically, since there is no temporal correspondence between the empirical and simulated FCD, it would be inappropriate to directly compute the Euclidean distance between them [26]. Instead, the difference between the empirical and simulated FCD matrices is quantified by the maximum distance between their cumulative distribution functions (CDFs), calculated using the Kolmogorov-Smirnov (KS) distance.

To minimize the objective function, Covariance Matrix Adaptation Evolution Strategy (CMA-ES) [54] was employed. During the training phase, CMA-ES was initialized randomly five times, with each initialization undergoing 100 iterations, resulting in a total of 500 candidate parameter sets. For each set of parameters, the simulation was repeated 20 times on the validation set, and the mean FC and FCD were computed from these 20 simulations, which were then used to optimize the objective function, ultimately yielding the best parameter set. The E/I ratio differences were calculated as:


$$\begin{array}{c} E/Idifference=\;E/I_{LSD}-E/I_{placebo} \end{array}$$ (12)

## Supporting information

S1 Fig PL states with the most significant group differences for k values ranging from 5 to 10. (TIF)

S2 Fig The Pearson correlation matrix of the PL states showing the most significant differences between the LSD and placebo groups at k values ranging from 5 to 10. (TIF)

S1 Table The Pearson correlation between each PL state and the 7 RSNs is presented as an array, where the first element represents the correlation coefficient (r) and the second element represents the FDR-adjusted p-value. P-values that reached statistical significance (<0.05) are highlighted in red.
(XLSX)

S2 Table FDR corrected p-values for the differences between the LSD and placebo conditions in terms of probability of occurrence, obtained from a permutation-based paired sample t-test. P-values that reached statistical significance (<0.05) are highlighted in red.
(XLSX)

S3 Table FDR corrected p-values for the differences between the LSD and placebo conditions in terms of dwell time, obtained from a permutation-based paired sample t-test. P-values that reached statistical significance (<0.05) are highlighted in red.
(XLSX)

S4 Table The Pearson correlation between the probability of occurrence of each PL state and the Kuramoto Order Parameter (OP) and the standard deviation of the OP (STD(OP)), where the FDR corrected p-values less than 0.05 are highlighted in red. (XLSX)

S5 Table P-values (uncorrected) and Cohen’s d for the differences between the LSD and placebo conditions in terms of the probability of transitioning from a given state to other states. The values are presented as tuples (p-value, Cohen’s d). P-values less than 0.05 are highlighted in red to indicate statistical significance.
(XLSX)

S6 Table P-values for the differences between PL states under LSD and placebo conditions in probability of occurrence and dwell time with k ranging from 2 to 10, with p-values less than 0.05 highlighted in red to indicate statistical significance. (XLSX)

S7 Table Pearson correlation between E/I ratio difference and 7 RSNs, with values in red indicating p-values less than 0.05 (FDR corrected). (XLSX)
